# Reproductive politics and women's empowerment; how does geopolitics control women?

**DOI:** 10.3389/fgwh.2025.1666773

**Published:** 2025-10-10

**Authors:** Gayathri Delanerolle, Sohier Elneil, Abirame Sivakumar, Om Kurmi, Vindya Pathiraja, David Ikwuka, Nhan Thi Nguyen, Nirmala Rathnayake, Peter Phiri, George Uchenna Eleje

**Affiliations:** 1 University of Birmingham, Birmingham, United Kingdom; 2 Hampshire and Isle of Wight Healthcare NHS Foundation Trust, Southampton, United Kingdom; 3 University College London, London, United Kingdom; 4 University of Jaffna, Jaffna, Sri Lanka; 5 Centre for Healthcare and Communities, Coventry University, Coventry, United Kingdom; 6 University of Ruhuna, Matara, Sri Lanka; 7 University of Rwanda, Kigali, Rwanda; 8 University of Medicine and Pharmacy, Ho Chi Minh City, Vietnam; 9 Nnamdi Azikiwe University, Awka, Nigeria

**Keywords:** reproductive politics, women's empowerment, geopolitics, abortion, child birth

## Abstract

Reproductive politics lie at the intersection of gender, power, and governance, shaping women's autonomy through laws, policies, and cultural norms. Historically, colonialism and population control initiatives marginalized women, particularly in the Global South, fostering distrust in healthcare systems. Feminist movements advocate for reproductive justice, yet economic and nationalistic interests continue to influence access to care. Governments regulate reproduction to control demographics, labor markets, and national power. Pronatalist and antinatalist policies, such as China's One-Child Policy, have led to coercive interventions, disproportionately affecting marginalized communities. Reproductive politics also shape masculinity, fatherhood, and state-controlled family structures. Global reproductive policies reflect ideological struggles, from restrictive abortion laws in Poland and the U.S. to progressive approaches in Nepal and Vietnam. Socioeconomic barriers further limit access to contraception, maternal healthcare, and fertility treatments. Achieving reproductive justice requires inclusive policies, healthcare reform, and recognition of reproductive rights as fundamental to gender equality.

## Introduction

Women's bodies have been the centre of politics for centuries due to reproductive health. Reproductive politics sit at the intersection of gender, power, and governance where women's reproductive autonomy is shaped by policies, laws, and cultural norms that reflect broader geopolitical strategies. Governments, religious institutions, and international organisations exert varying degrees of control over reproductive rights, shaping not only individual choices but also national and global power dynamics. Whilst feminist movements have long advocated for reproductive justice, the intersection of nationalism, economic interests and ideological agendas continue to shape access to reproductive healthcare, often at the expense of women's empowerment. This commentary explores how reproductive politics serve as both a site of oppression and a tool for women's empowerment, highlighting the role of geopolitical control in shaping reproductive freedoms and constraint.

Historical forces, such as colonialism and population program profoundly shaped the geopolitics of women's health. Colonial regimes imposed by western medical systems often disregarded the autonomy of indigenous women, laying the foundations for health disparities seen today. During the mid-20th century, global population control initiatives backed by governments and institutions targeted the Global South with a variety of non-consensual contraceptive interventions, framing reproductive health through a coercive lens of demographic threat rather than rights or care. The interventions influenced, and somewhat exacerbated social constructs in relation to gender, race and cast systems leading to contemporary reproductive health policies that made the public mistrust healthcare system. These legacies continue to affect women's health with the lack of dedicated service provisions to cover complex population requirements and limited funding.

Geopolitical control refers to the ways in which states, governments, and global powers exercise authority over populations by shaping policies, laws, and resources in alignment with their strategic, economic, or ideological interests. In the context of reproduction, it captures how reproductive rights, healthcare access, and demographic outcomes are influenced or restricted as instruments of national security, labor management, cultural dominance, or international power relations (1, 2).

### Reproductive politics and masculinity: the unseen constraints

Reproductive politics extend beyond the realm of women's bodies, shaping masculinity, fatherhood, and state-controlled family structures. Yet, despite the significant ways in which reproductive policies impact men, the primary terrain of regulation has historically been women's bodies. This asymmetry is not incidental; it is rooted in deeply entrenched socio-political hierarchies that define fertility, motherhood, and reproduction as central to national identity, economic stability, and demographic anxieties.

As Yuval-Davis (3) observed, women are often positioned as “integrational transmitters of cultural traditions, customs, songs, cuisine, and, of course, the mother tongue.” This symbolic role extends beyond mere cultural continuity, it reinforces reproductive hierarchies, where women's fertility is not just a private concern but a matter of national importance. In many societies, the capacity to bear children becomes a political issue, entrenching patriarchal structures that dictate who should reproduce, when, and under what conditions (3).

Fatherhood as a national imperative to assist declining birth rates is another aspect where men are increasingly encouraged to embrace traditional family roles. Japan's declining fertility crisis, for instance, has prompted state-led efforts to promote work-life balance for men in hopes of increasing family formation (4). However, these efforts often fall short, as the economic realities of insecure employment and the pressures of masculinity create barriers to active fatherhood.

### Reproductive gatekeeping

**Figure 1:**
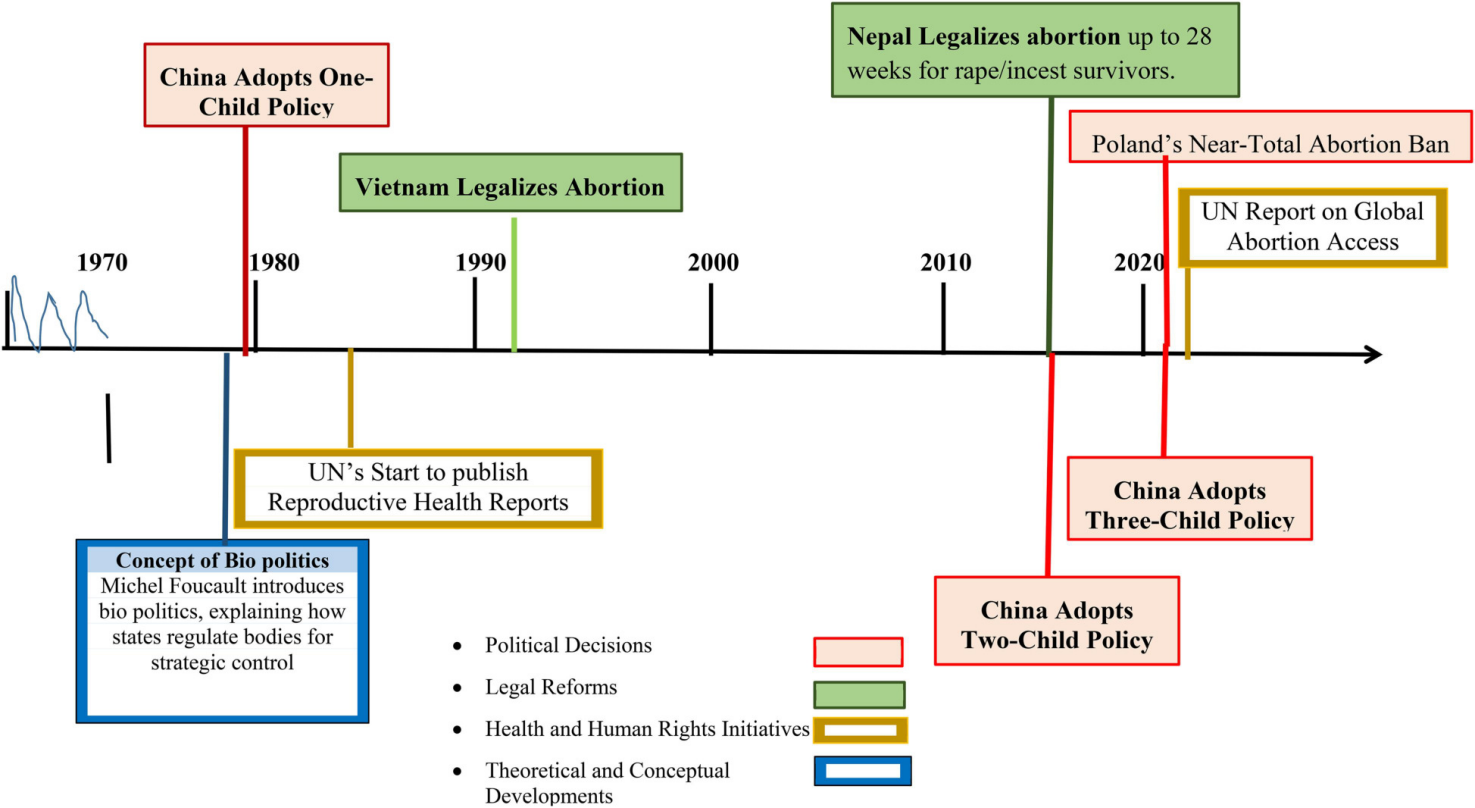
Geopolitical decisions which made significant differences in the history.

Historically, states have regulated reproduction as a tool to shape demographic trends, labour markets, and national power structures (Figure 1). Reproductive governance is deeply shaped by localised socio-cultural norms, legal frameworks, and systemic biases, with Lesbian, Gay, Bisexual, Transgender, Queer+ (LGBTQ+) and transgender individuals facing unique and often overlooked barriers**.** While some countries have progressive reproductive policies**,** they may still exclude trans and non-binary people due to rigid cis-normative definitions of reproductive health. For example, in Latin America, despite growing LGBTQ+ rights movements, health systems often fail to accommodate transmasculine individuals seeking abortion or fertility services. In parts of Africa and Asia**,** where colonial-era sodomy laws and cultural stigmas persist, LGBTQ+ individuals face heightened reproductive health discrimination, forced sterilization, or denial of care. Even in high-income countries, trans and non-binary people encounter structural barriers, including misgendering, lack of inclusive medical guidelines, and legal obstacles to parental recognition. Ignoring these regional disparities risks erasing the reproductive needs of LGBTQ+ individuals and reinforcing policies that fail to address their specific vulnerabilities.

## Methodology

### Study design

This paper employed an integrative review–commentary methodology, appropriate for synthesising diverse forms of evidence across disciplines. Unlike systematic reviews that focus narrowly on empirical outcomes, an integrative review allows incorporation of theoretical, historical, and policy materials, providing the breadth necessary to examine reproductive politics within geopolitical and intersectional frameworks.

### Data sources and search strategy

Relevant literature was identified through purposive searches of PubMed, Science Direct, and interdisciplinary databases, alongside grey literature repositories from the World Health Organisation (WHO), World Bank, and United Nations (UN). The search covered the period 1970–2025 to capture both historical colonial influences and contemporary reproductive governance debates.

### Inclusion and exclusion principles

We included (i) peer-reviewed articles on reproductive politics, governance, and rights; (ii) landmark international policy texts; and (iii) feminist theoretical and historical analyses foundational to the field. Sources were excluded if they were non-scholarly, lacked empirical or policy relevance, or did not address intersections between geopolitics and reproductive health.

### Data analysis

A contextual and narrative synthesis approach was adopted. Materials were critically read and coded for recurring themes related to colonial legacies, geopolitical influence, reproductive governance, and intersectional inequities. Evidence was then synthesised narratively to highlight convergences and divergences across regions and disciplines. This approach enabled integration of policy, theory, and empirical evidence into a coherent analysis, foregrounding the structural determinants of reproductive rights and their implications for global health governance.

The politics of reproduction manifest in various ways, including:

- *Pronatalist Policies*: Governments experiencing declining birth rates often implement measures to encourage childbirth. Hungary, for example, offers financial incentives and tax benefits to larger families, while Russia celebrates *Mother's Day* with awards for women who bear multiple children (5, 6). These policies reinforce gendered expectations, where women's reproductive capacities are leveraged for national goals.
- *Antinatalist Policies:* In contrast, some states have aggressively pursued population control. China's *one-child policy* (1979–2015) exemplifies state intervention in reproduction, leading to forced sterilizations and selective abortions (7–9). Similarly, forced sterilization campaigns have targeted marginalized groups, such as Indigenous women in the United States and Peru, underscoring how reproductive regulation is often weaponized against vulnerable populations (10).
- *Reproductive Surveillance*: The collection and use of reproductive data have become tools for managing populations. In some cases, reproductive technologies are used to track and control the fertility of specific groups, often disproportionately affecting women from lower socioeconomic backgrounds, refugees, and ethnic minorities (11, 12). This surveillance operates under the guise of public health but frequently reproduces existing social inequalities.

### Geopolitics and regulation of reproduction

Reproductive health has never existed in isolation from power, rather it has been deeply engrained with political agendas, public policies, and ideological control, shaping the lives of women and the global populous. As Kligman (57) observed, “*state policy and ideological control are experienced in everyday life*” This perspective echoes Michel Foucault's (1978) concept of *biopolitics*, wherein the state regulates bodies and populations through policies that appear neutral but serve strategic interests (58, 61). From population control programs to restrictions on abortion, reproductive governance has historically been a means by which states exert authority over life itself. China's One-Child Policy (1979–2015) is one of the most well-documented examples of reproductive governance, reflecting how states regulate bodies and populations under the guise of neutrality while serving strategic interests. At the time of implementation, China faced concerns of rapid population growth and perceived this to be a potential hindrance to economic modernisation. The One-Child Policy was formally introduced in 1979 by Deng Xiaoping's government as a drastic population control measure. The policy was enforced through a combination of legal mandates, economic penalties, and coercive tactics, disproportionately affecting rural and marginalized communities. The government imposed strict reproductive surveillance through birth quotas, mandatory contraception, and forced sterilizations (7). Women were required to obtain official permission before becoming pregnant, and local officials monitored compliance. The policy was enforced through economic sanctions, employment discrimination, and social stigma for families that violated birth limits. In more extreme cases, there were reports of forced abortions and sterilisations to maintain compliance, particularly in rural areas (13). Recognising the economic and demographic consequences of the One-Child Policy, including an aging population, shrinking workforce, and declining birth rates, China gradually relaxed its reproductive policies where a relaxation in 2013 allowed couples to have a second child if one parent was an only child followed by the introduction of a two-child policy in 2015. In 2021, in response to population decline, China introduces a three-child policy and extended maternity leave.

The political construction of the “ideal” mother is another aspect of injustice. Not all women are equally encouraged to reproduce. In many Western nations, immigrant and minority women often face reproductive surveillance, where policies are designed to control the fertility of certain populations. The forced sterilisation of Indigenous women in Canada (as recently as the 2010s) highlights how reproductive politics can be used to maintain racial and cultural hierarchies (14).

The United Nations has addressed the issue of reproductive control through abortion laws in various reports. One notable example is the UN's Abortion Policies and Reproductive Health around the World report, which examines how different legal frameworks impact women's reproductive autonomy and health outcomes. Abortion laws are deeply entangled in geopolitical dynamics, shaping and reflecting power structures, global health priorities, and ideological divides. The impact of these laws on women's health is multifaceted, influencing access to healthcare, reproductive autonomy, and international relations. Restrictive abortion laws, particularly in low- and middle-income countries (LMICs), are often tied to historical colonial legacies, religious influences, and foreign policy pressures (15). Countries with liberal abortion laws such as Canada and Sweden have positioned themselves as global leaders in reproductive rights, actively funding international reproductive health programs (16). In contrast, conservative governments, particularly under the U.S. Global Gag Rule, have cut funding for organizations that provide abortion services, widening health disparities by limiting access to safe procedures (17).

### Maternal healthcare, fertility treatments, and contraception access

The intersection of maternal healthcare, fertility treatments, and contraception access is a critical domain in modern public health, with profound implications for both individual well-being and societal progress (18). As we navigate the complexities of reproductive health, it becomes increasingly evident that disparities in access to these essential services remain a persistent challenge. Together, these disparities illustrate how structural inequities, policy gaps, and sociocultural factors converge to restrict reproductive justice. Addressing these challenges requires integrated policy responses, investment in healthcare systems, and a reframing of reproductive health as a fundamental right rather than a privilege determined by geography or class.

### Maternal healthcare: a foundation for reproductive health

Maternal healthcare is the cornerstone of a healthy pregnancy and childbirth experience (19). Global efforts to reduce maternal morbidity and mortality have made significant strides in recent decades, with improved prenatal care, skilled birth attendance, and timely interventions being key factors in reducing preventable deaths. However, gaps in access to quality maternal care persist, particularly in low- and middle-income countries, where financial, geographic, and cultural barriers continue to affect women's access to essential services (20, 21). Maternal healthcare continues to be uneven across regions, with preventable maternal morbidity and mortality disproportionately affecting women in low- and middle-income countries due to inadequate infrastructure, limited skilled personnel, and financial barriers (22, 23).

In high-income countries, while maternal mortality rates have generally declined, disparities in outcomes based on race, ethnicity, and socioeconomic status remain troubling. Studies have shown that marginalized communities, particularly Black and Indigenous women, face a disproportionately high risk of complications and death during pregnancy and childbirth (24). Structural inequities, such as systemic racism and unequal access to healthcare, exacerbate these outcomes, calling for targeted efforts to ensure that maternal care is both accessible and equitable for all women.

#### Fertility treatments: expanding options, raising ethical concerns

Even though the landscape of reproductive health and family-building has evolved significantly, yet the policies and laws governing adoption and surrogacy remain frustratingly outdated, even in progressive nations like the UK (25). As medical advancements continue to offer hope to individuals and couples struggling with infertility, legislative frameworks must keep pace to ensure equitable access to parenthood.

Access to fertility treatments, such as *in vitro* fertilization, remains highly stratified, often available only to wealthier populations or those living in urban centers, thereby reinforcing socioeconomic inequities in the ability to build families (26, 27). Similarly, contraception access is shaped not only by availability and affordability but also by cultural norms, religious restrictions, and policy environments that can either empower women or reinforce gatekeeping mechanisms over their reproductive choices (28, 29).

A significant yet often overlooked group affected by these archaic policies includes women with menstrual disorders, such as endometriosis and polycystic ovary syndrome (PCOS). Many of these women require assisted reproductive technologies (ART) like *in vitro* fertilization (IVF) to conceive. However, IVF is only a viable option for those who can safely carry a pregnancy (30). For others, surrogacy becomes a necessary alternative. Unfortunately, legal barriers, restrictive eligibility criteria, and a lack of standardized policies often make surrogacy an inaccessible or complex process. Fertility treatments have revolutionized reproductive possibilities, offering hope to couples and individuals facing infertility challenges. Technologies like IVF have made it possible for many to achieve parenthood who might otherwise have been unable to conceive. However, the high cost, limited accessibility, and the emotionally and physically demanding nature of these treatments continue to create barriers for many.

As the demand for fertility services rises, it is critical that we consider the ethical and social implications of these advancements. The rapid pace of innovation in reproductive technologies raises questions about the regulation and oversight of practices like embryo selection and genetic screening, which have the potential to lead to new forms of inequality or discrimination (31). Furthermore, while fertility treatments can benefit individuals of various backgrounds, the growing commodification of fertility, and the emphasis on age-related fertility decline, risks perpetuating societal pressures and stigmas surrounding reproduction.

Equally important is the consideration of access to fertility treatments across socioeconomic strata. The exorbitant costs of IVF and related technologies mean that only a limited segment of the population can afford these services, often leaving lower-income women and marginalized communities at a disadvantage. Governments and healthcare providers must prioritise efforts to make fertility treatments more accessible and to address the financial and logistical barriers that prevent many from receiving care.

Beyond medical necessity, adoption and surrogacy policies must also account for the diverse realities of modern family structures. LGBTQ+ individuals and transgender people seeking to become parents face additional layers of legal and societal challenges (32). Many adoption agencies and surrogacy frameworks still operate under outdated biases that exclude or disadvantage non-heteronormative families. As a result, individuals who could provide loving, stable homes encounter unnecessary obstacles rooted in outdated norms rather than contemporary medical or ethical considerations.

The pressing question remains: why are laws governing reproductive rights and family-building still stuck in the past when medical advancements and societal shifts demand reform? A re-evaluation of surrogacy and adoption policies is essential to ensure that all individuals, regardless of gender identity, sexual orientation, or medical condition, have equitable access to parenthood. Policymakers must recognize that reproductive rights extend beyond biological capabilities and should prioritize inclusivity, medical feasibility, and ethical considerations in shaping the future of family-building.

#### Contraception access: empowering choice, addressing inequality

Contraception is a fundamental pillar of reproductive health and autonomy (33), allowing individuals to make informed choices about their reproductive lives. Access to contraception has been transformative for women's health, education, and economic participation, enabling women to plan pregnancies and participate fully in the workforce and society. Despite the widespread availability of contraceptive methods, barriers to access, whether financial, political, or cultural, continue to impede full equity in contraception use.

For many women, particularly in rural or underserved areas, the lack of access to reliable contraception can lead to unintended pregnancies (34), which can, in turn, affect educational and economic outcomes. In some regions, contraceptive methods are subject to political and ideological opposition, resulting in reduced availability and even criminalization of certain methods, such as emergency contraception and abortion services. Furthermore, access to contraception for marginalized groups, including adolescents, low-income women, and women of color, is often fraught with barriers such as discrimination, stigma, and a lack of culturally sensitive care.

Innovative policy measures, such as the expansion of family planning services and the provision of free or subsidized contraceptive options, have demonstrated success in increasing access and empowering individuals to make reproductive choices. However, there is still much to be done to ensure that contraception is universally available and accessible, without restriction, in all parts of the world.

Advances in healthcare technologies, including reproductive health, must be coupled with policies that reduce disparities in access to services. This includes advocating for healthcare systems that are inclusive, affordable, and responsive to the needs of all individuals, regardless of their socio-economic or demographic background (35). Additionally, increasing public awareness, education, and access to accurate information about reproductive health will empower individuals to make informed choices about their health and well-being.

As we look to the future, it is critical that we engage in ongoing conversations about the ethical, social, and economic implications of reproductive healthcare. Governments, healthcare providers, and communities must work collaboratively to create a more equitable landscape for reproductive health, ensuring that all women and individuals have the resources, support, and opportunities they need to make choices that are right for their lives.

In nations where religious conservatism and right-wing populism hold political influence, reproductive policies often prioritise foetal rights over women's bodily autonomy. Poland and the United States provide two stark examples of how abortion restrictions function as mechanisms of ideological control.

- Poland's Near-Total Abortion Ban (2021): Poland has some of the most stringent abortion laws in Europe, shaped largely by the influence of the Catholic Church and nationalist politics (36). The 2020 Constitutional Tribunal ruling**,** which banned abortion even in cases of foetal abnormalities, reflects a broader political project that merges religious doctrine with state power. By restricting reproductive rights, the government enforces a vision of Polish identity rooted in conservative, patriarchal values, where women's primary role is as mothers and bearers of national continuity (37). The law has sparked mass protests under the banner of the *Strajk Kobiet* (Women's Strike), revealing how reproductive control remains a highly contested political issue.
- The U.S. Post-Roe Landscape (2022–Present): The overturning of Roe v. Wade (1973) in the Dobbs vs. Jackson Women's Health Organization decision (2022) has similarly reshaped reproductive rights in the United States. In conservative-led states, abortion bans and foetal personhood laws reinforce a broader political effort to reassert patriarchal control over women's bodies, often couched in the language of “family values” (38). Scholars argue that these restrictions disproportionately impact marginalised communities, including low-income women, racial minorities, and immigrants, who face barriers to out-of-state travel for legal abortions (39). The rollback of reproductive rights in the U.S. aligns with a global authoritarian trend**,** where nationalist and populist movements seek to reassert control over gender and sexuality as part of their broader political agenda (40).
- In Nepal, abortion following sexual assault is legally permitted under the Safe Motherhood and Reproductive Health Rights Act 2018, which allows termination up to 28 weeks of gestation in cases of rape or incest. This aligns with Nepal's commitment to reproductive rights and gender justice, as reflected in its' Constitution (2015), which guarantees women's rights to safe abortion. Despite progressive laws, social stigma, lack of awareness, and barriers to accessing services, particularly in rural areas, hinder survivors from seeking timely care. The influence of patriarchal norms and victim-blaming attitudes often discourages reporting and reinforces silence around sexual violence and abortion.
- Vietnam has one of the most liberal abortion laws in Southeast Asia. Under the 1989 Law on Protection of People's Health, abortion is legal on request during the first 22 weeks of pregnancy with no restrictions based on reason; however, abortion is strictly prohibited for reasons of gender selection. The country follows a public health approach, offering abortion services through state-run hospitals and reproductive health clinics, making it widely accessible. To guarantee for women'health, the Vietnamese Ministry of Health issued the Decision no. 4620/ QĐ-BYT in November 25, 2009 called “National Guidelines on Reproductive Health Care Services” in which regulates on safe abortion and medical facilities must meet technical and equipment requirements to perform safe abortions. In cases of sexual assault, Vietnamese law does not impose additional restrictions on abortion beyond the gestational limit. However, social stigma, gender norms, and lack of legal support for survivors can create barriers to access. Many victims face shame, discrimination, and fear of social repercussions, deterring them from seeking both justice and reproductive healthcare. While services exist, rural areas often have limited access, and survivors may struggle with financial and emotional burdens due to inadequate state-supported counselling and legal aid.
- Similar to many countries in South Asia, Abortion in Sri Lanka is highly restricted under the Penal Code of 1883, permitted only to save the mother's life. It remains illegal in cases of rape, incest, fetal abnormalities, or maternal health risks. Attempts to reform the law, including a 2017 proposal to allow abortion in limited cases, faced strong opposition from religious and conservative groups and were not passed. Despite legal restrictions, an estimated 600–700 unsafe abortions occur daily, leading to serious health risks. Cultural and religious beliefs, particularly Buddhism's stance on the sanctity of life, contribute to the resistance against legalization. Advocacy for contraceptive access, comprehensive sexuality education, and limited decriminalization continues amid ongoing public health concern.

#### Women's health as a human rights & diplomatic issue

International organizations such as the United Nations, WHO, and global feminist movements emphasize that safe and legal abortion is a fundamental human right (41). However, some governments use nationalist or religious justifications to resist global norms, reinforcing domestic restrictions on abortion. For example, Poland's near-total abortion ban in 2020 sparked international criticism from the European Parliament and human rights bodies, highlighting how abortion laws can fuel diplomatic tensions (42).

#### Influence of transnational funding & policies

Foreign aid policies play a crucial role in shaping reproductive healthcare access worldwide. The Mexico City Policy, also known as the Global Gag Rule, has significantly affected abortion access in Africa, Latin America, and Asia by withdrawing U.S. funding from organizations that offer abortion-related services (43). Meanwhile, China and Russia have engaged in geopolitical maneuvering by influencing development assistance policies in LMICs, sometimes reinforcing restrictive reproductive health laws rather than supporting progressive reforms (44).

#### Feminist resistance & policy reforms

Despite restrictive policies in many regions, grassroots feminist activism has successfully pushed for policy changes, proving the power of transnational solidarity. Movements such as Argentina's Green Wave and Ireland's pro-choice campaign have led to historic abortion law reforms (45). Additionally, regional bodies such as the European Union exert pressure on member states to uphold progressive reproductive health policies, though resistance remains in countries like Poland and Hungary (46).

Despite these inequities, transnational feminist movements have successfully challenged restrictive reproductive policies and called for decolonisation of global health governance. For example, Argentina's Green Wave movement (2020) successfully fought for the legalization of abortion despite long-standing neocolonial population control narratives (59, 60). African feminist health networks have developed locally-led reproductive health programs to reduce dependency on foreign aid, such as MamaYe in Nigeria and Fòs Feminista in Latin America. Indigenous and Black women-led organisations in HICs are pushing for reproductive justice frameworks that address historical reproductive oppression, including forced sterilisations and racial disparities in maternal health (47). Similarly, Phiri and colleagues (48) offers valuable insights that can be decolonised reproductive health. The authors emphasize that decolonization extends beyond mere inclusivity; it requires re-examining history through the experiences of those most affected by colonization. This perspective is crucial in reproductive health, where Western-centric models often dominate, potentially marginalizing indigenous knowledge and practices. Phiri et al. critique superficial changes, such as adding diverse authors to syllabuses, arguing for a comprehensive reevaluation of Westernized histories and teachings. Applying this to reproductive health means acknowledging and integrating non-Western reproductive practices and beliefs, thereby challenging embedded systemic biases.

Grassroots and community-led movements play a pivotal role in resisting restrictive reproductive policies, challenging state and global power structures, and shaping reproductive justice frameworks. These movements emerge in response to state-imposed reproductive control, colonial legacies, and global health inequities, often bridging local activism with transnational solidarity networks. For example;

- Latin America: The Green Wave Movement: The Marea Verde (Green Wave) movement has been a driving force behind legalizing abortion in Latin America. Originating in Argentina, the movement mobilized feminists, healthcare workers, and legal advocates to push for the landmark legalization of abortion in 2020 (49). Through mass protests, legal advocacy, and social media campaigns, activists successfully shifted public and political opinion. The movement's momentum extended beyond Argentina, influencing Mexico, Colombia, and Chile, where similar efforts have led to significant policy changes. One of the key successes of the Green Wave has been its intersectional approach, incorporating Indigenous and working-class women's perspectives, ensuring that reproductive justice is not only about legal access but also about healthcare equity. This strategy has challenged state authority over reproductive governance and countered the influence of conservative religious groups that have historically opposed abortion rights.
- Africa: Decolonizing Reproductive Health Through Indigenous Advocacy: In many African countries, restrictive abortion laws and colonial-era reproductive policies continue to shape health governance (50). However, grassroots organizations such as Fòs Feminista (operating in Latin America and Africa) and the TICAH initiative in Kenya have emerged to provide community-driven reproductive healthcare solutions. These initiatives focus on legal advocacy to challenge colonial-era abortion laws, particularly in Kenya and Nigeria, where access remains limited despite growing feminist activism. Another critical strategy involves empowering traditional birth attendants and midwives, integrating culturally relevant reproductive health solutions into formal healthcare systems. In some regions, these groups also provide LGBTQ+ reproductive health services, which remain a highly stigmatized and underfunded area of healthcare (51).
- Eastern Europe: Resistance Against Rising Anti-Choice Policies: In Poland, where abortion laws are among the strictest in Europe, feminist grassroots movements have led massive street protests and underground abortion networks. The “Black Protest” (Czarny Protest) and Aborcyjny Dream Team have provided abortion pills and safe access to services, despite legal crackdowns (52). Poland's near-total abortion ban, implemented in 2020, led to a transnational feminist response, with organizations like Women on Waves offering cross-border abortion assistance. These activists have leveraged digital activism, medical tourism, and underground networks to ensure reproductive autonomy, even as the Polish government intensifies restrictions. This movement highlights the geopolitical nature of reproductive rights, as Poland's anti-choice policies align with broader nationalist and conservative agendas seen in Hungary and other parts of Eastern Europe.
- LGBTQ+ and Trans-Inclusive Reproductive Activism; Mainstream reproductive justice movements have historically excluded trans and non-binary individuals, but in recent years, organizations such as Trans Health Initiative (Thailand), Queer Trans Mutual Aid (U.K.), and the TGEU network in Europe have worked to expand reproductive healthcare access for gender-diverse individuals (53).These groups focus on ensuring gender-affirming reproductive healthcare, including trans pregnancy care and access to hormone therapies. Another critical area of activism has been fighting against forced sterilization policies, which remain legal in several European countries as a requirement for legal gender recognition. Additionally, trans-inclusive legal advocacy seeks to redefine reproductive rights beyond cisnormative frameworks, ensuring that abortion and fertility services are available to all individuals regardless of gender identity

### Neocolonialism and global reproductive inequities

Global health interventions, whilst often positioned as mechanisms of empowerment, can also function as instruments of neocolonial control (48). Western-led reproductive health initiatives in the Global South, for example have fraught history of coercion, population control and imposition of western norms. Reproductive inequities manifest in disproportionate maternal mortality rates, lack of contraceptive access, and unsafe abortion practices, particularly in LMICs. The World Health Organization (54) estimates that 94% of maternal deaths occur in LMICs, where restrictive abortion laws and underfunded healthcare systems limit reproductive choices. From forced sterilisation programs in India and Peru to the unethical trials of contraceptives in African nations, reproductive healthcare has been shaped by racialised and economic hierarchies that prioritise geopolitical interests over bodily autonomy. Even contemporary efforts such as promotion of long-acting reversible contraceptives in low income countries, raise ethical concerns when implemented without robust frameworks for informed consent and reproductive choice.

Neocolonialism operates through economic dependencies, foreign aid policies, and institutional power structures, where HICs maintain control over LMIC reproductive healthcare systems. One of the most glaring examples is the U.S. Global Gag Rule, which prohibits foreign organizations receiving U.S. funding from providing or promoting abortion services. This policy has led to increased maternal mortality and unsafe abortions in Africa, Latin America, and parts of Asia (55). Similarly, pharmaceutical companies and research institutions in HICsoften conduct clinical trials in LMICs without guaranteeing access to the developed medical innovations for local populations (56). HICs also dictate population control narratives, which have historically influenced family planning programs in LMICs. For example, structural adjustment programs (SAPs) imposed by the International Monetary Fund (IMF) and World Bank in the 1980s and 1990s led to the privatization of healthcare in many LMICs, reducing access to reproductive services for marginalised populations (35).

### The paradox of women's empowerment

While reproductive rights are often framed as key to women's empowerment, their implementation is frequently dictated by political expediency than genuine commitment to gender equity. The rise of femonationalism- where feminist rhetoric is co-opted to justify anti-immigration policies and Islamophobic discourses-illustrates how reproductive rights can be mobilised for restricting Muslim women's access to reproductive healthcare, coupled with policies that demonise migrant fertility, underscore the ways in which reproductive governance serves nationalist projects than feminist liberation.

### Towards and decolonial and international approach

To truly advance reproductive justice, policies must be informed by an intersectional and decolonial lens that centres the lived experiences of marginalised communities. This means moving beyond pro-choice/pro-life binaries to address structural determinants of reproductive oppression, including economic precarity, healthcare accessibility, and racial and gender discrimination. It also requires challenging the geopolitical structures that instrumentalise women's bodies for political and economic ends to prevent further biases in reproductive healthcare that leads to tangible inequalities from maternal mortality disparities to barriers in accessing fertility treatments for LGBTQ+ individuals, as well preventing *conditional reproductive care* as not all women experience “*choice*” in the same way.

Ultimately, reproductive politics is not merely about individual choice but the power structures that shape and constrain those choices. Women's empowerment cannot be achieved through top-down policies that perpetuate control under the guise of protection, rather, it must emerge from grassroots, community led movements that prioritise reproductive autonomy as a fundamental human right that becomes part of everyday lived experiences. Only through such an approach can we dismantle geopolitical forces that regulate, exploit, and commodify women's bodies in the name of national and global interest.

## Education for women

Education plays a crucial role in shaping women's empowerment, yet its accessibility is often influenced by geopolitical control and reproductive politics. Governments and global institutions dictate policies on women's reproductive rights, affecting their autonomy over family planning, healthcare, and economic participation. In many regions, restrictive abortion laws, limited contraceptive access, and cultural norms hinder women's ability to complete education and enter the workforce. Geopolitical power dynamics such as foreign aid, religious influence, and global policies often determine how countries legislate on women's health and rights. Nations with progressive reproductive policies tend to have higher female literacy rates, while those imposing strict reproductive controls see lower education levels due to early pregnancies, forced marriages, and gendered economic dependency. Thus, education, reproductive rights, and geopolitical control are deeply interconnected, shaping women's opportunities and their role in society.

### Future research and policy debates

The findings underscore the need for future research that situates reproductive politics within intersecting structures of gender, class, race, and colonial legacies. Comparative cross-national studies could deepen understanding of how state policies differently regulate reproduction across political systems, while longitudinal approaches would clarify the long-term impacts of coercive interventions on women's health, autonomy, and intergenerational trust in healthcare institutions. Future scholarship should also explore men's roles in reproductive politics, particularly how masculinities and fatherhood are shaped by state regulation, and how these dynamics reinforce or challenge gendered hierarchies. Moreover, ethnographic and participatory methods would be valuable in centering women's lived experiences especially from marginalized groups within global policy debates.

On the policy front, the manuscript highlights critical tensions that demand urgent attention. Governments must reconcile demographic and economic interests with the recognition of reproductive rights as inalienable human rights. Policy debates should shift from population control to reproductive justice, prioritizing equitable access to contraception, maternal healthcare, fertility treatments, and safe abortion services. This necessitates integrating reproductive health into broader gender equality and human rights agendas, ensuring that reforms address socioeconomic disparities that disproportionately exclude women in the Global South and minority communities in the Global North. International agencies and donors must also be held accountable for the colonial continuities embedded in global reproductive governance, promoting transparency, community participation, and culturally responsive healthcare delivery.

By linking empirical inquiry with policy reform, future work can help transform reproductive politics from a mechanism of control into a framework for empowerment and justice.

## Limitations

This study is limited by its reliance on secondary sources and its cross-sectional scope, which cannot fully capture temporal shifts or community-level adaptations. The lack of dedicated funding for global women's health research also constrains the depth and reach of analyses such as this. Funders should therefore reconsider the importance of investing in research that is both globally relevant and locally relatable, ensuring that diverse populations are meaningfully represented in reproductive health policy debates.

## Conclusion

Reproductive governance is shaped by local socio-cultural norms, political economies, and enforcement mechanisms, generating marked disparities in reproductive health outcomes. Legal frameworks may suggest uniformity, yet lived experiences reveal persistent inequities, whether in Latin America's restrictive abortion regimes alongside feminist advocacy, Africa's colonial legacies and religious influences, or systemic barriers affecting racialised and marginalised groups in high-income countries. To reduce these inequities, reforms must go beyond legal provisions to strengthen equitable enforcement, embed culturally informed care, and prioritise reproductive justice within health systems. Comparative policy analyses and longitudinal assessments are needed to examine how reproductive governance evolves across contexts and over time. Such evidence would enable more responsive, equitable, and sustainable interventions that address both structural inequities and lived realities.
